# A prospective, randomized, double-blind, placebo-controlled trial of acute postoperative pain treatment using opioid analgesics with intravenous ibuprofen after radical cervical cancer surgery

**DOI:** 10.1038/s41598-018-28428-4

**Published:** 2018-07-05

**Authors:** Xintong Liu, Xifan Wang, Wenshuai Zhao, Lanying Wei, Pengjiao Zhang, Fei Han

**Affiliations:** Department of Anesthesiology, The third Affiliated Hospital, Harbin Medical University, Harbin, Heilongjiang 150081 China

## Abstract

This study assessed the efficacy and tolerability of intravenous ibuprofen in the improvement of post-operative pain control and the reduction of opioid usage. Patients were randomly divided into placebo, ibuprofen 400 mg and ibuprofen 800 mg groups. All patients received patient-controlled intravenous morphine analgesia after surgery. The first dose of study drugs was administered intravenously 30 min before the end of surgery and then every 6 hours, for a total of 8 doses after surgery. The primary endpoint of this study was the mean amount of morphine used during the first 24 hours after surgery. Morphine use was reduced significantly in the ibuprofen 800 mg group compared with the placebo group (P = 0.04). Tramadol use was reduced significantly in the ibuprofen 400 mg and ibuprofen 800 mg groups compared with the placebo group (P < 0.01). The area under the curve of visual analog scale pain ratings was not different between groups. Safety assessments and side effects were not different between the three groups. Intravenous ibuprofen 800 mg was associated with a significant reduction in morphine requirements, and it was generally well tolerated for postoperative pain management in patients undergoing radical cervical cancer surgery.

## Introduction

Postoperative pain in the recovery process remains a challenge for anesthesiologists. Approximately 80% of patients experience postoperative pain, and over 30% of patients experience severe pain^1,2^. Undertreated postoperative moderate-to-severe pain is associated with an increased risk for progression to a chronic painful state. Undertreated postoperative pain increases the risk of myocardial ischemia, impairs rehabilitation and wound healing, delays gastrointestinal motility, alters immune responses, and increases pulmonary complications due to the poor respiratory effort and thromboembolism due to immobilization^3–6^.

Opioids are widely used in the treatment of acute postoperative pain. Over 60% of patients who experience moderate or severe postoperative pain received morphine as a postoperative pain therapy^7,8^. However, the failure of acute postoperative pain treatment may be the result of the use of opioid monotherapy^9^. Opioids cause respiratory depression, sedation, drowsiness, pruritus, skin rash, urinary retention, delayed gastrointestinal motility, and postoperative vomiting and nausea^10^. The concept of “multimodal analgesia” was proposed in the early 1990s to overcome these opioid-induced adverse effects and achieve sufficient analgesia^11,12^. The efficacy and safety of intravenous (IV) ibuprofen as a multimodal approach to pain management was investigated in different clinical trials, and it is accepted as an adjunct to opioids for acute postoperative pain management^13–16^. The purpose of this study was to assess the efficacy and tolerability of different doses of IV ibuprofen in the improvement of pain control and the reduction of opioid use in patients undergoing radical cervical cancer surgery.

## Methods

This study was a prospective, randomized, double-blind, placebo-controlled trial. This study was performed at the Third Affiliated Hospital, Harbin Medical University and the ethics committee of the Third Affiliated Hospital, Harbin Medical University approved this study. This study was registered in the Chinese Clinical Trial Registry (Registration number: ChiCTR-IOR-16009101, date of registration: 26/08/2016). All methods were performed in accordance with the relevant guidelines and regulations. Informed consent forms and all amendments were reviewed and approved by the ethics committee of the Third Affiliated Hospital, Harbin Medical University, before any study-specific screening procedures were performed and any preoperative medications were administered.

### Participants

The study population consisted of 60 female patients who were scheduled for radical cervical cancer surgery and were expected to require postoperative hospitalization and patient-controlled IV analgesia (PCIA) of morphine for at least 24 hours. Included patients were 18 to 70 years old with American Society of Anesthesiologists (ASA) physical status I-III and the ability to reliably provide self-reports of pain. Patients were excluded from the study if they had a history of allergy or hypersensitivity to ibuprofen, aspirin, COX-2 inhibitors or other non-steroidal anti-inflammatory drugs (NSAIDs), or a history of tolerance or dependence to narcotics or opioids. Patients with Hb <90 g/L, weight <40 kg, a history of asthma or heart failure, or pregnant or nursing were also excluded. Patients were not eligible if they had a platelet count less than 80,000/mm3, gastrointestinal bleeding history within the previous 6 weeks, a history of bleeding diathesis, or a recent history or increased risk of intracerebral hemorrhage. Patients with alanine aminotransferase or aspartate aminotransferase levels over 1.5 times the normal upper limit or creatinine over the normal upper limit were not eligible. Patients were excluded if they were taking warfarin, lithium, or a combination of angiotensin-converting enzyme inhibitors and furosemide. Patients were excluded if they received any analgesic, muscle relaxant, or sedative medications within 24 hours of study medication administration, excluding the sedatives or muscle relaxants that were used during the surgical procedure. Local anesthesia, nerve blocks, epidural anesthesia and analgesia were not allowed during the pre/intra-operative periods.

### Study Design

All patients were assigned in a 1:1:1 ratio using a double-blind, simple randomization scheme to receive IV placebo, ibuprofen 400 mg, or ibuprofen 800 mg every 6 hours for a total of 8 doses in the first 48 hours of the study. Investigators, patients, and care providers were blinded to intervention assignment. Only the study pharmacist was unblinded.

Anesthesia methods were standardized in all three groups. Anesthesia was induced using 2 μg/kg remifentanil, 0.05 mg/kg midazolam, 1–2 mg/kg propofol, and 0.6 mg/kg rocuronium. A tracheal catheter was inserted after 2 min of rocuronium administration. The entire course of anesthesia was maintained using 4–8 mg/kg/h propofol and 5–12 µg/kg/h remifentanil, which sustained changes in blood pressure within 20% of the initial levels and bispectral index (BIS) between 40 and 60 during surgery. Ventilation rate was 12/min. Tidal volume was adjusted to 6–10 ml/kg to maintain end-tidal CO_2_ at 35–45 mmHg.

Morphine (100 mg) was diluted in 200 ml of 0.9% saline and added to the PCIA pump. The PCIA pump was set at a loading dose of 1.5 mg, a background infusion of 0.5 mg/h, a 1 mg bolus, and a lockout interval of 5 min. All patients received PCIA pump infusions 30 min before the end of surgery.

**Figure 1:**
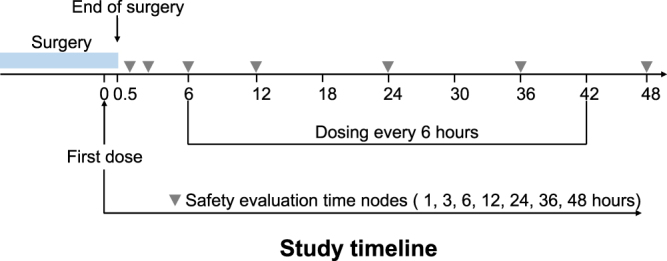
Experimental protocol.

The first dose of IV ibuprofen or placebo was administered 30 min before the end of surgery (Fig. 1). Subsequent doses of ibuprofen or placebo were administered every 6 hours over the next 48 hours. Remedial analgesia, tramadol 100 mg, IV, was given during the study observation phase in patients who complained of pain after three bolus infusions of PCIA.

### Efficacy Assessment

The primary endpoint of this study was the mean amount of morphine used during the first 24 hours after surgery. Secondary endpoints included tramadol requirement (as additional remedial analgesia during the study period) and the mean change in pain intensity. Pain intensity at rest and with movement were assessed using patient self-reporting with a 100-mm visual analog scale (VAS, 0 = no pain to 100 = intense pain), the Bruggemann comfort scale (BCS, 4 = no pain, 0 = intense pain) and revised overall pain performance scale (OPPS, 0 = no pain, 12 = intense pain)^17^. VAS was recorded at 1, 3, 6, 12, 24, 36 and 48 hours after administration of the first dose of study medication. The area under curve (AUC) of VAS up to 24 hours after administration of the first dose of study medication was analyzed between groups. BCS and OPPS were evaluated at 1, 3, 6, 12, 24, 36 and 48 hours after administration of the first dose of study medication.

### Safety Assessment

Tolerability and safety were evaluated as adverse events, vital signs, and laboratory assessments of patients in all three groups. Adverse events, including pyrexia, headache, dizziness, cough, postoperative inflammation, epigastric pain, pruritus, nausea, vomiting, time of exhaust, gastrointestinal bleeding, urinary tract infection, and concomitant medications were recorded and compared until day 4 of the study. Vital signs, including blood pressure, heart rate, respiratory rate, and body temperature were recorded at 1, 3, 6, 12, 24, 36 and 48 hours after administration of the first dose of study medication. Laboratory assessments included biochemistry, blood routine, blood coagulation function (prothrombin time, activated partial thromboplastin time, plasma fibrinogen and plasma D-dimer), urinalysis, and electrocardiogram 24–48 hours after administration of the last dose of study medication.

### Statistical Analysis

Sample size calculation was according to morphine use of the pilot study. Initial power calculations indicated that 14 patients of each group would provide 80% power to show a reduction in morphine use between the placebo group and the ibuprofen 800 mg group at an α level of 0.05. Assuming a dropout rate of approximately 10%, 16 patients in each group should be enrolled.

Quantitative variables are presented as the means ± standard deviation (SD). Level variable are presented as interquartile range. Categorical variables are presented as percentages. The general characteristics of the patients, VAS scores, the AUC of VAS, the morphine dose, and tramadol dose were analyzed using ANOVA followed by the LSD test. BCS and OPPS scores were analyzed using a Kruskal-Wallis H test and Mann-Whitney U test. Chi-square and Fisher’s exact test were used to compare differences in side effects and the number of patients with abnormal laboratory assessments between groups. P < 0.05 was considered statistically significant. All statistics were assessed using SPSS 22.0.

## Results

**Figure 2:**
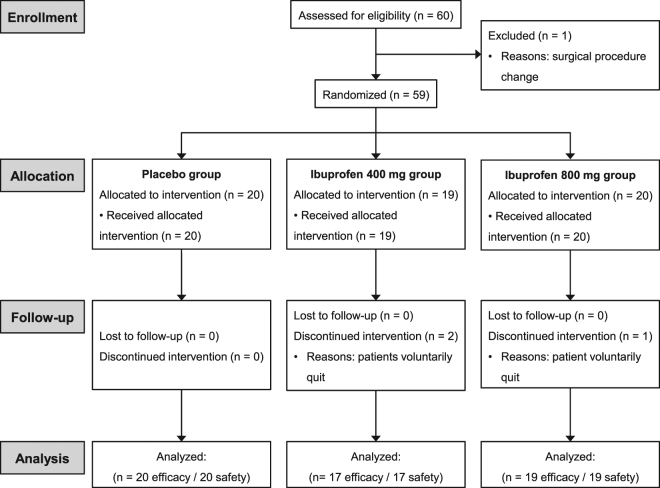
CONSORT diagram.

Within 60 patients, one patient was excluded due to surgical procedure change and 3 patients withdrew consent (Fig. 2). A total of 56 patients, 20 patients in the placebo group, 17 patients in the ibuprofen 400 mg group, and 19 patients in the ibuprofen 800 mg group, were enrolled for analysis. Baseline demographic characteristics were not significantly different between the three groups. There was no difference in age between the placebo (45.6 ± 5.3 years), ibuprofen 400 mg (45.9 ± 7.4 years), and ibuprofen 800 mg (45.2 ± 8.6 years) groups. There were no differences in weight between the placebo (56.0 ± 8.0 kg), ibuprofen 400 mg (60.3 ± 9.6 kg), and ibuprofen 800 mg (58.7 ± 10.8 kg) groups.

### Primary efficacy variable

**Figure 3:**
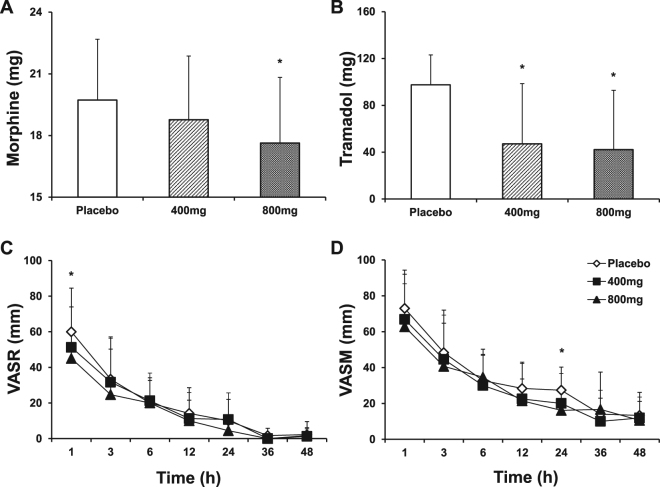
Morphine and tramadol use during the 24 hour postoperative period and VAS at rest and with movement during 48 hours postoperatively. (**A**) morphine dose. (**B**) tramadol dose. (**C**) VAS at rest. (**D**) VAS with movement. *P < 0.05, vs. placebo. VAS, visual analog scale; VASR, visual analog scale at rest; VASM, visual analog scale with movement.

Morphine use was significantly reduced during the first 24 hours in the ibuprofen 800 mg group (17.6 ± 3.2 mg) compared with the placebo group (19.7 ± 3.0 mg, P = 0.04, Fig. 3A). The morphine use between the placebo and ibuprofen 400 mg (18.8 ± 3.1 mg) groups and between the ibuprofen 400 mg and 800 mg groups was not significantly different.

### Secondary efficacy variables

Tramadol use was significantly reduced in the ibuprofen 400 mg (47.1 ± 51.4 mg) and ibuprofen 800 mg (42.1 ± 50.7 mg) groups compared to the placebo group (97.5 ± 25.5 mg) during the first 24 hours after the surgery (P < 0.01, Fig. 3B). Tramadol use was not different between the ibuprofen 400 mg and ibuprofen 800 mg groups.

Based on VAS evaluation, the patients of the ibuprofen 800 mg group was associated with significant reductions in pain intensity at rest compared with the patients of the placebo group at 1 hour after administration of the first dose of study medication (P = 0.049, Fig. 3C). The ibuprofen 800 mg group was also associated with significant reductions in pain with movement compared with the placebo group at 24 hours after administration of the first dose of study medication (P = 0.04, Fig. 3D). The pain intensity at rest or with movement at other time points was no significant difference between three groups. The AUCs of VAS at rest or with movement in the placebo, ibuprofen 400 mg, and ibuprofen 800 mg groups were not significantly different (Table 1).

**Table 1:** Pain measured as the area under the curve of VAS (mm*h).

	Placebo (n = 20)	Ibuprofen 400 mg (n = 17)	Ibuprofen 800 mg (n = 19)
1–24 h (at rest)			
Mean (SD)	505.00 (482.07)	308.75 (208.10)	290.83 (284.93)
Median	270.00	352.50	247.50
Min	135.00	40.00	0.00
Max	1320.00	490.00	680.00
P value, vs. Placebo		0.419	0.331
1–24 h (with movement)			
Mean (SD)	807.00 (556.67)	665.00 (438.38)	543.33 (338.78)
Median	530.00	650.00	507.50
Min	400.00	145.00	0.00
Max	1765.00	1125.00	930.00
P value, vs. Placebo		0.660	0.250
1–6 h (at rest)			
Mean (SD)	164.37 (97.55)	152.50 (124.26)	124.00 (95.96)
Median	165.00	167.50	125.00
Min	15.00	20.00	0.00
Max	360.00	400.00	270.00
P value, vs. Placebo		0.532	0.427
1–6 h (with movement)			
Mean (SD)	228.12 (97.24)	216.25 (115.71)	191.50 (106.79)
Median	222.50	230.00	177.50
Min	100.00	40.00	0.00
Max	400.00	400.00	370.00
P value, vs. Placebo		0.613	0.504

**Figure 4:**
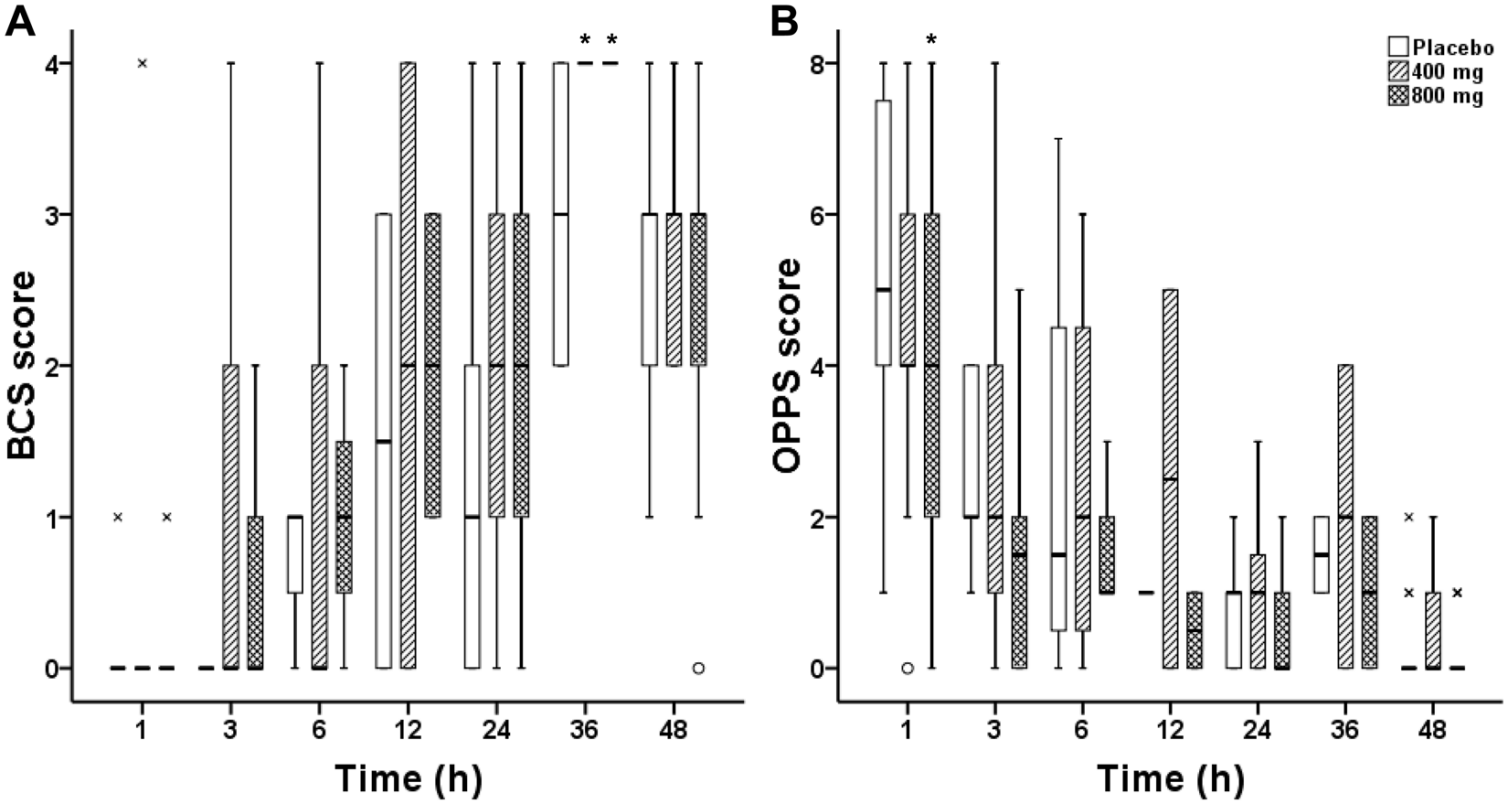
BCS score and OPPS score during 48 hours postoperatively. (**A**) BCS score. (**B**) OPPS score. × represents the highest value. ○ represents an outlier. *P < 0.05, vs. placebo. BCS, bruggemann comfort scale; OPPS, overall pain performance scale.

BCS scores in the ibuprofen 400 mg and 800 mg groups were significantly higher than the placebo group at 36 hours after administration of the first dose of study medication (P = 0.03, Fig. 4A). OPPS scores in the placebo group were significantly higher than the ibuprofen 800 mg group at 1 hour after administration of the first dose of study medication (P = 0.02, Fig. 4B).

### Safety Analysis

The safety assessment of the three groups over the course of the study were shown in Table 2. There were no differences in the incidence of pyrexia, headache, dizziness, cough, postoperative inflammation or epigastric pain between groups. The incidence of pruritus, nausea, vomiting, abnormal exhaust time and gastrointestinal bleeding were not different between groups. No significant differences in liver or renal function were found between groups. No hemoglobin decrease or coagulation disorders were found in any group. There were no significant difference in blood pressure, heart rate, respiratory rate, or body temperature between groups during the study period. There were no acute cardiovascular or severe acute events in any group.

**Table 2:** Safety assessments in the three groups.

	Placebo (n = 20)	Ibuprofen 400 mg (n = 17)	Ibuprofen 800 mg (n = 19)	P
General disorders				
Pyrexia	18 (90%)	14 (82%)	14 (74%)	0.413
Headache and dizziness	1 (5%)	2 (12%)	1 (5%)	0.675
Cough	6 (30%)	7 (41%)	10 (53%)	0.357
Postoperative inflammation	1 (5%)	2 (12%)	1 (5%)	0.675
Epigastric pain	1 (5%)	5 (29%)	1 (5%)	0.041*
Skin and subcutaneous tissue disorders				
Pruritus	3 (15%)	2 (12%)	1 (5%)	0.608
Gastrointestinal disorders				
Nausea	7 (35%)	5 (29%)	2 (11%)	0.186
Vomiting	2 (10%)	1 (6%)	0	0.38
Exhaust within 48 h	1 (5%)	4 (24%)	1 (5%)	0.123
Gastrointestinal bleeding	0	0	0	>0.999
Liver disorders				
Alanine aminotransferase increased	0	0	0	>0.999
Aspartate aminotransferase increased	0	0	0	>0.999
Renal and urinary disorders				
Urinary tract infection	7 (35%)	3 (18%)	2 (11%)	0.159
Blood urea nitrogen increased	0	0	0	>0.999
Creatinine increased	0	0	0	>0.999
Clinical laboratory assessments				
Hemoglobin decreased	1 (5%)	1 (6%)	1 (5%)	0.993
Prothrombin time extended	0	0	0	>0.999
Activated partial thromboplastin time	0	0	1 (5%)	0.371
extended	2 (10%)	2 (12%)	2 (11%)	0.985
Plasma fibrinogen increased	1 (5%)	0	0	0.400
Plasma D-dimer increased				

^*^Recalculated by Fisher’s exact test. P = 0.059, placebo group vs. ibuprofen 400 mg group; P = 0.744, placebo group vs. ibuprofen 800 mg group; P = 0.067, ibuprofen 400 mg group vs. ibuprofen 800 mg group.

## Discussion

Postoperative pain continues to be a considerable problem despite advances in our understanding of the mechanisms involved. A patient’s unwillingness to use opioids may be one reason for inadequate pain relief. Concern about side effects is a main contributor to this reluctance^18^. The use of single morphine analgesia greatly increased the incidence of side effects, such as intractable nausea, vomiting, respiratory issues and emesis, which hinders perfectly effective postoperative pain management. The use of additional medications to reduce the need for opioids is widely endorsed^19–21^. Various analgesics act at different sites in the nervous system, which results in synergistic analgesia and a reduction in the side effects of the sole agent^11^.

Opioids block only the perception of pain, and the anti-inflammatory activity of ibuprofen helps prevent and alleviate the tissue inflammation that causes pain. Oral ibuprofen has been widely used as a safe and effective treatment for pain, fever, and inflammation for more than 30 years^22,23^. Oral ibuprofen effectively blocks pain and inflammation, in part via the prevention of prostaglandin production^24^. An IV formulation of ibuprofen may arrest the inflammatory cascade triggered by invasive procedures, reduce or prevent the development of postoperative pain, and avert the sensitization of pain receptors. However, there are some safety concerns to NSAIDs use. The gastrointestinal and renal toxicity and general bleeding risks are increased with NSAIDs use^25^. However, many of these effects are associated with longer-term use^26^. An IV ibuprofen preparation would most likely be used on a short-term basis in hospitalized patients and in out-patient surgical procedures, which would decrease the incidence of these safety concerns. Some studies examined investigational formulations of IV ibuprofen, and none of these studies demonstrated any safety concerns^27–29^. These studies used a range of ibuprofen doses up to 800 mg and found no renal issues, gastrointestinal toxicity or bleeding, and no effect on transfusion requirements or hemoglobin levels.

The current study evaluated the safety and efficacy of IV ibuprofen-assisted postoperative analgesia in patients undergoing radical cervical cancer surgery. Evidence from epidemiological studies suggests that women undergoing hysterectomy may be particularly at risk for adverse pain experiences^30^. The strategy presented in this study involving IV ibuprofen as an adjunct to morphine may prove beneficial for these patients. The results of this study suggest that the use of IV ibuprofen at a dose of 800 mg, but not 400 mg, every 6 hours alleviated postoperative pain and was significantly morphine-sparing with a reduction in morphine use in patients undergoing radical cervical cancer surgery. This use of ibuprofen may reduce some of the well-established adverse events associated with opioid analgesia. IV ibuprofen 800 mg significantly reduced pain at rest and with movement in postoperative pain management^13,15,16^. A meta-analysis of 17 randomized controlled trials, comprised of 400 patients who received opioid analgesia plus an NSAID for the management of postoperative pain and 389 patients who received monotherapy with opioid analgesia, demonstrated that patients who received both medications consumed fewer opioids and had lower pain scores than patients who received opioid monotherapy^20^. No difference in the incidence of adverse effects was observed between opioids and opioids plus NSAIDs groups^20^. Our results are consistent with these reports. There was no significant difference in the incidence of gastrointestinal bleeding, toxicity, renal toxicity, or generalized bleeding between the ibuprofen 400 mg, ibuprofen 800 mg, and placebo groups. The findings of this study on IV ibuprofen at different doses further add to the body of evidence supporting the effectiveness of multimodal analgesic regimens for the management of acute postoperative pain^31–33^.

One limitation of the study was the small number of patients, which may reduce the statistical power of the results. However, the main results of this study are consistent with previous studies^13–16^. The current study was the first trial to focus on patients undergoing radical cervical cancer surgery, which provides evidence to physicians in evaluating the safety and efficacy of IV ibuprofen-assisted postoperative analgesia in these patients.

In conclusion, the findings of this study suggest that postoperative IV injections of ibuprofen 800 mg, but not 400 mg, was associated with significant reductions in morphine requirements for postoperative pain management, which may reduce some of the well-established adverse events associated with opioid analgesia in patients after surgery. There was no significant difference in the incidence of gastrointestinal bleeding, toxicity, renal toxicity, generalized bleeding or other side effects between the ibuprofen 400 mg, ibuprofen 800 mg, and placebo groups. IV ibuprofen was generally well tolerated for postoperative pain management.
